# Deep learning-based forecasting of electricity consumption

**DOI:** 10.1038/s41598-024-56602-4

**Published:** 2024-03-18

**Authors:** Momina Qureshi, Masood Ahmad Arbab, Sadaqat ur Rehman

**Affiliations:** 1 Department of Computer Systems Engineering, University of Engineering and Technology Peshawar, Peshawar, Pakistan; 2 School of Sciences Engineering and Environment University of Salford, Manchester, UK

**Keywords:** BEMS, LSTM, Electricity demand forecasting, Anomaly detection, Energy consumption, Future forecasting, Model optimizer, Energy science and technology, Engineering

## Abstract

Building energy management systems (BEMS) are integrated computerized systems that track and manage the energy use of many pieces of building-related machinery and equipment, including lighting, power systems, and HVAC systems. Modern buildings must have BEMSs in order to reduce energy usage while maintaining comfort. Not only for energy-saving purposes, BEMS is essential in enhancing the quality of the energy supply, which helps to gain a better understanding of how energy is used and the building's energy usage. When the dynamics of a building's energy usage are known, it is possible to determine which changes are most likely to reduce consumption. Numerous connected devices, operating modes, energy usage, and environmental factors can all be monitored and controlled in real-time using BEMS. Changing operating times and setting points to maximize comfort and efficiency is made simple by this. In this paper, we have primarily addressed the two significant issues of model optimization and electricity consumption forecasts. Future forecasting has been done using the LSTM based time series approach. We generated data on the amount of electricity consumed by a hospital facility and tested our suggested methodologies on actual data. The findings gained demonstrated that the strategies were successful with both types of data. On actual data, the trend in electricity consumption can be accurately predicted. Several model optimizers enhanced the suggested methods' performance as well. Our objective function gain accuracy result of 95%.

## Introduction

When the buildings are in operation, human error and technical malfunctions cause a lot of energy to be lost. Any energy-saving technique that has buildings as its primary objective has a major impact on overall energy use and carbon emissions. In order to monitor and detect anomalous usage and alert building managers to execute suitable cost-saving measures, smart building facilities are now a complement to Building Energy Monitoring Systems (BEMS). As a result, this project suggests a deep neural network-based model for forecasting energy use.

A Building Energy Management System (BEMS) is a collection of hardware and software technologies designed to assist organizations in tracking, managing, and optimizing their buildings' energy use. Heating, ventilation, and air conditioning (HVAC), lighting, and other energy-consuming equipment are just a few of the systems that BEMS can monitor and manage in a building. BEMS evaluate and optimize the energy use of buildings using data from sensors, meters, and other sources. Building owners and facility managers can make data-driven decisions to increase energy efficiency since BEMS gives them access to real-time information on energy consumption.

Initiatives to improve energy efficiency in the building industry can significantly contribute to the development of a greener future. Furthermore, the Building Automation System (BAS)’s massive collection of building operating data has made it possible to create data-driven methods for predicting energy use. Due to the recent growth of sensor devices, modern buildings today contain an increasing number of sensors and smart meters. The building manager is informed if consumption patterns are discovered through data analysis from these devices that do not match the typical profiles, and the necessary energy-saving actions could then be implemented. More importantly, early detection and notification of unusual behavior, such as gas leaks, may help to prevent potentially deadly catastrophes for safety–critical building services, such as gas consumption. Additionally, it is difficult to identify unusual consumption patterns due to the volume and rate of sensor data generation. The goal of this work is to suggest a method for capturing historical consumption trends that uses unsupervised learning with unlabeled data. By assuming that historical data are predominantly normal, the framework builds a model representing normal consumption behavior, after which this model is used to identify new patterns.

Therefore, the need for improved monitoring technologies that can automatically identify consumption patterns and provide precise information about the variables involved is what drove this work. Energy managers and energy service providers lack the time to assess energy consumption and hunt for anomalies. Instead, BEMS should have these features to quickly recognize and report such instances. The concept was put into practice, and the advantages of this approach were highlighted, using energy data from several commercial buildings that had been obtained from online sources. Most of the current research in this area has been limited to techniques that have been proposed within specific application domains. For symbolic sequences, most of the existing techniques either focus on detecting anomalies in data obtained in the domain of system call intrusion detection or in the domain of biological sequences. For univariate time series data, especially in the field building energy, the research is even sparser.

The goal of the study is to close the gap between existing research on deep neural network-based energy forecasting in time series data and algorithm optimization. Based on this knowledge, the objectives of this project are:

1. To develop a prediction model of time-series electricity consumption data using LSTM network method.
2. To optimize the algorithm through several methods of deep learning for increasing the performance of the proposed algorithms.

## Literature review

The International Energy Outlook 2019 by the (U.S. Energy Information Administration, 2019) reported that global energy consumption has anticipated an increase of nearly 50% between 2018 and 2050. The building sector recorded 30–45% of the total energy demand globally and is among the largest contributors compared to industrial and transportation^1^. Furthermore^2^, suggested that growth in population and income as well as increasing need for comfort will result in the progressive rise in energy demand.

Energy efficiency and savings methods have been a top concern for energy policies in most nations due to the growth of energy consumption and carbon dioxide (CO2) emissions in the built environment^3^. Commercial structures, especially office and academic structures, are among those with the highest energy usage^4^.

As a result, several government initiatives to reduce energy use have turned their attention to the commercial building sector^5^. According to earlier studies^6^ and an on-site study of existing university buildings^4^, modern office buildings have a large potential for energy savings. This potential is in the range of 6–29%.

An essential component of this study is the analysis of time series data. Time series data refers to anything that is measured over a period of time, and its primary use is to identify trends and patterns that can be used to anticipate and draw conclusions about future outcomes. For instance^7^, noted that it is crucial for the electricity function to model load forecasts in an effective and safe manner. Additionally, it's critical to generate accurate projections and operating rebuilding costs that are as low as possible^8^.

In addition to that, power system planners and demand controllers could guarantee that there would be a sufficient supply of electricity to meet growing demands by forecasting future load demands^9^. Due to these factors, load forecasting has drawn organisations and researchers with a similar interest in predicting energy use using a variety of methodologies, from traditional methods to cutting-edge methods.

Mocanu et al.^10^ divided electricity demand forecasting into three categories based on the time horizon of prediction: short-term load forecasts (STLF) covering a time period between one hour and one week, medium-term load forecasts (MTLF) covering a time period between one week and one year, and long-term load forecasts (LTLF) covering a time period of more than one year. According to^11,12^, and^13^, short-term forecasts are typically helpful for scheduling generation capacity and short-term maintenance, evaluating short-term energy storage usage, as well as real-time control of building energy systems and optimising fuel purchase plans.

On the other hand, choices regarding the installation of new distributed generation and storage systems as well as the creation of effective demand response techniques^10^ are made using medium- to long-term projections^14^. Planning and trading on energy markets at the regional level may benefit from anticipating aggregated electricity usage over the medium- to long-term^15^ (Table 1).

**Table 1:** Different algorithms used for forecasting electricity consumption.

References	algorithm/method	Year	Results
^9^	Forecasting future load demands	2000	Suggest for future forecasting and Electricity consumption analysis
^10^	STLF, MTLF, LTLF Time Division	2016	Predict short, Medium, Long term forecast
^11,12^ and^13^	short-term forecasts helpful for scheduling generation capacity and short-term maintenance and short-term forecasts	2015, 2016	Short-term maintenance forecast prediction
This Study	LSTM and future electricity consumption	2023–2024	Model optimization and electricity consumption forecasts using LSTM. Objective Function give accuracy of 95%

In summary, researchers are now concentrating on discovering methods that could model their data and produce an accurate forecast in accordance with their application and limits, whether it be short-, medium-, or long-term load projections.

## Methodology

### Overview

The following sections discusses the detailed methodology process. The paper has four main parts. The first part discusses the details of how the datasets were set up. In the second parts, the overall forecasting of electricity consumption algorithm is explained in details respectively. It covers the working of LSTM and future electricity consumption. Part three covers the optimization of algorithm that was used in conjunction with the overall system. In this part various optimizers and hyper parameters are used in order to get optimal result.

### Dataset

A real-world electricity demand dataset was used in this project that was downloaded from open-source data portal OpenEI. The dataset has a year records of hourly electricity consumption of a hospital building located in Phoenix, USA. The readings were taken in a year from January 1st, to December 31st, starting from 00:00 to 23:00 each day that sums up to a total of 8760 data instances. The data was arranged in a time series manner whereby the power consumption in kilo watt hour (kWh) had been measured in an hour resolution.

**Figure 1:**
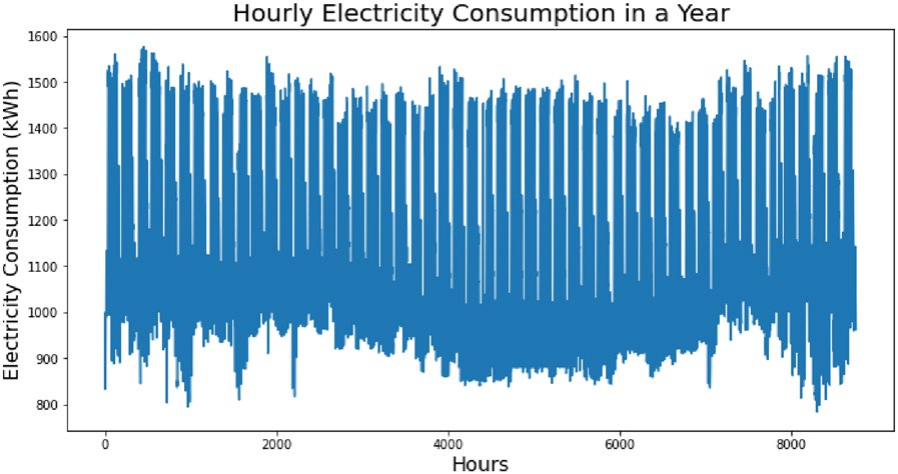
The electricity load profile of dataset for the 8760 h.

Figure 1 shows the entire 8760 time-steps of electricity consumption data plot taken at hourly intervals in a year. The magnitude is the electricity consumption measured in kWh at any given hour.

The monthly average values of electricity consumption for each month are displayed in Table 2.

**Table 2:** Average electricity consumption.

Jan	Feb	Mar	Apr	May	June	July	Aug	Sept	Oct	Nov	Dec
1189.23	1166.03	1190.21	1182.77	1163.78	1137.14	1111.31	1132.36	1114.51	1143.15	1191.02	1155.29

In the dataset, the monthly electricity consumption is showing a regular weekdays-weekend pattern in which higher consumption readings were recorded during the weekdays while lower readings were recorded in the weekends. From the figure of daily average consumption, we can see that in certain days, the minimum hourly reading marked lower than the rest of the days. A daily and weekly trend that is like actual electricity consumption is shown by the exploratory analysis of the dataset.

### LSTM network for prediction

**Figure 2:**
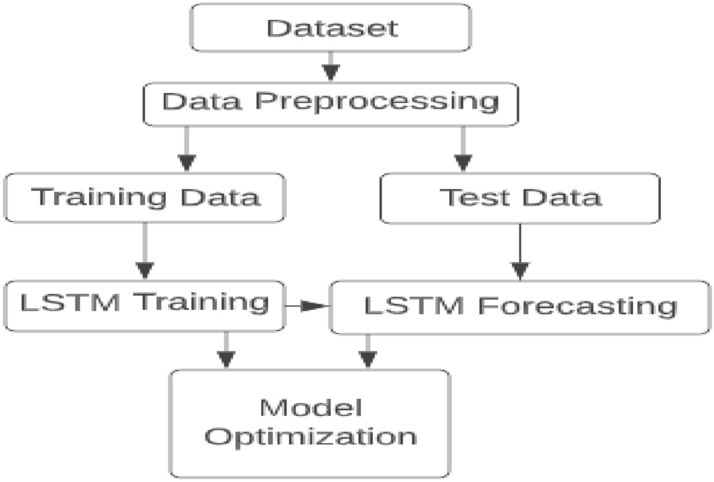
Proposed Framework for the Forecasting Model.

The primary goal of this study was to use an LSTM regression network to forecast future electricity use. The proposed framework for this section of the system is shown in block form in Fig. 2, along with input and output modules, data pre-processing, an LSTM network, and a module for model optimization. The anticipated power consumption sequence data for a period of time is the output data while the input data is the sequence for a certain period of predicted electricity consumption.

LSTM algorithm is particularly built to avoid the problem of long term dependency. Although some other machine learning algorithms can also process time series data; however, those algorithms cannot manage time series data with huge delay because of the disappearance of gradient. The LSTM is an advanced neural network algorithm that has practically the default behaviors to remember information for long periods of time^16^. LSTM regression network was defined prior being trained. The model was implemented in TensorFlow Keras in Python which provides a high-level API for implementation of Deep Neural Networks. The main objective of the LSTM for a given time series is to find a function f as in Eq. (1):

1 $$ {\text{Y}}_{{\text{t}}} = {\text{f}}\left( {{\text{Y}}_{{{\text{t}}{ - }{1}}} ,{\text{Y}}_{{{\text{t}}{ - }{2}}} , \ldots ,{\text{Y}}_{{{\text{t}}{ - }{\text{p}}}} } \right) $$

This calculates a function that, using p lags of the same energy consumption, explains the current energy consumption data. For the LSTM model to begin learning, we transformed the time series into X and Y matrices. Then, we reorganized our data into a format that an LSTM algorithm could use as input. It has the form of [samples, time-steps, input-features] and a 3-dimensional tensor.

The time step is the point of observation in the samples, which is equivalent to the amount of time steps we run our recurrent neural network. In our case, each data sample input into LSTM algorithm represented sixty time steps and contained a single feature of electricity consumption at that time step. We normalized the data as a pre-processing step in a range between 0 and 1 making the mean of data as zero and standard deviation as one. The data standardization is necessary to prevent the training from diverging and make the model better fit. In this algorithm 90% of the data was used in model training and 10% of the data was used as test set for model evaluation.

### Model optimization

#### Objective function

Our objective function is to maximize the R-squared and conversely minimize the mean absolute loss. R-squared is also called coefficient of determination which is a measuring metrics for a supervised regression algorithm that measures how well our regression line fits our data. It is the percentage of the dependent feature's variance that the independent feature explains.

An R-squared value of 1 shows a best fit, and an R-squared value of 0 shows a failure in modelling the data by the algorithm^17^.

$$ R^{2} = 1 - \frac{{SS_{RES} }}{{SS_{TOT} }} = 1 - \frac{{\sum\nolimits_{i} {\left( {y_{i} - \hat{y}_{i} } \right)^{2} } }}{{\sum\nolimits_{i} {\left( {y_{i} - \overline{y}_{i} } \right)} }} $$

#### Optimizer

The weights of each epoch are changed when a deep neural network model is being trained in order to reduce training loss. In a neural network, the optimizer function is in charge of changing the weights and learning rate attributes, which produces a maximum overall accuracy and a minimal loss. Millions of parameters are typically present in deep neural network models. As a result, choosing the appropriate weights is a difficult assignment for the model. The neural network model's weights and learning rate can be updated using a variety of optimizers. The program will determine the best fit optimizer to use, though. The optimizer is used to speed the deep learning training process.

#### Hyper parameters

Since many hyper-parameters affect how well a neural network performs, designing one is a difficult task that mostly depends on the depth of the designer's experience. The following levels correlate to the hyper-parameters that were taken into consideration when creating an effective neural network structure.

Level I: The number of neurons in hidden layers.

The performance of neural networks is significantly influenced by the number of neurons in the hidden layers.

Level II: The number of hidden layers.

The number of concealed layers is a second hyper-parameter that must be taken into account and is also crucial. The range of representative hidden layer numbers was set to 1 to 10. As the neural network's depth increased, its abilities grew stronger. However, if the network contains too many layers, it could lead to overfitting.

Level III: Activation function.

The challenges discussed in this work that cannot be solved by a linear function are solved by adding nonlinear elements using an activation function. In the experiment, several activation functions were selected for our regression model.

Based on the foregoing, it can be concluded that the hyper-parameters have a combined impact on the neural network's performance and cannot be considered independently. The starting point for our work was the optimization phase's settings. Till we obtained a minimum loss in the network, the model was further tweaked. In addition to these hyper parameters, we selected a number of epochs as the training duration by conducting numerous experiments. Dropout and other variables were also used and given appropriate values.

## Experiment and results

### Overview

This section presents the conducted experiments and all the outcomes of the experiments. In Section "LSTM for Forecasting" includes the results of the performance when running the LSTM forecasting algorithms.

### LSTM for forecasting

#### Model training

The training of LSTM model for forecasting future power consumption was done in a setting, that is, 90% of the dataset was trained and then tested on the last 10%. The training set was further split in training and validation set that was helpful and used as a preventive measure against over fitting. Several optimizers that have been specified in the algorithm in several experiments for optimizing the algorithm, are tabulated in Table 3.

**Table 3:** Optimizers used in several settings.

Setting	Optimizer
Setting1	SOG
Setting2	Nadam
Setting3	Adadelta
Setting4	Ftrl
Setting5	RMSprop
Setting6	Adam

#### Forecast future power consumption

**Figure 3:**
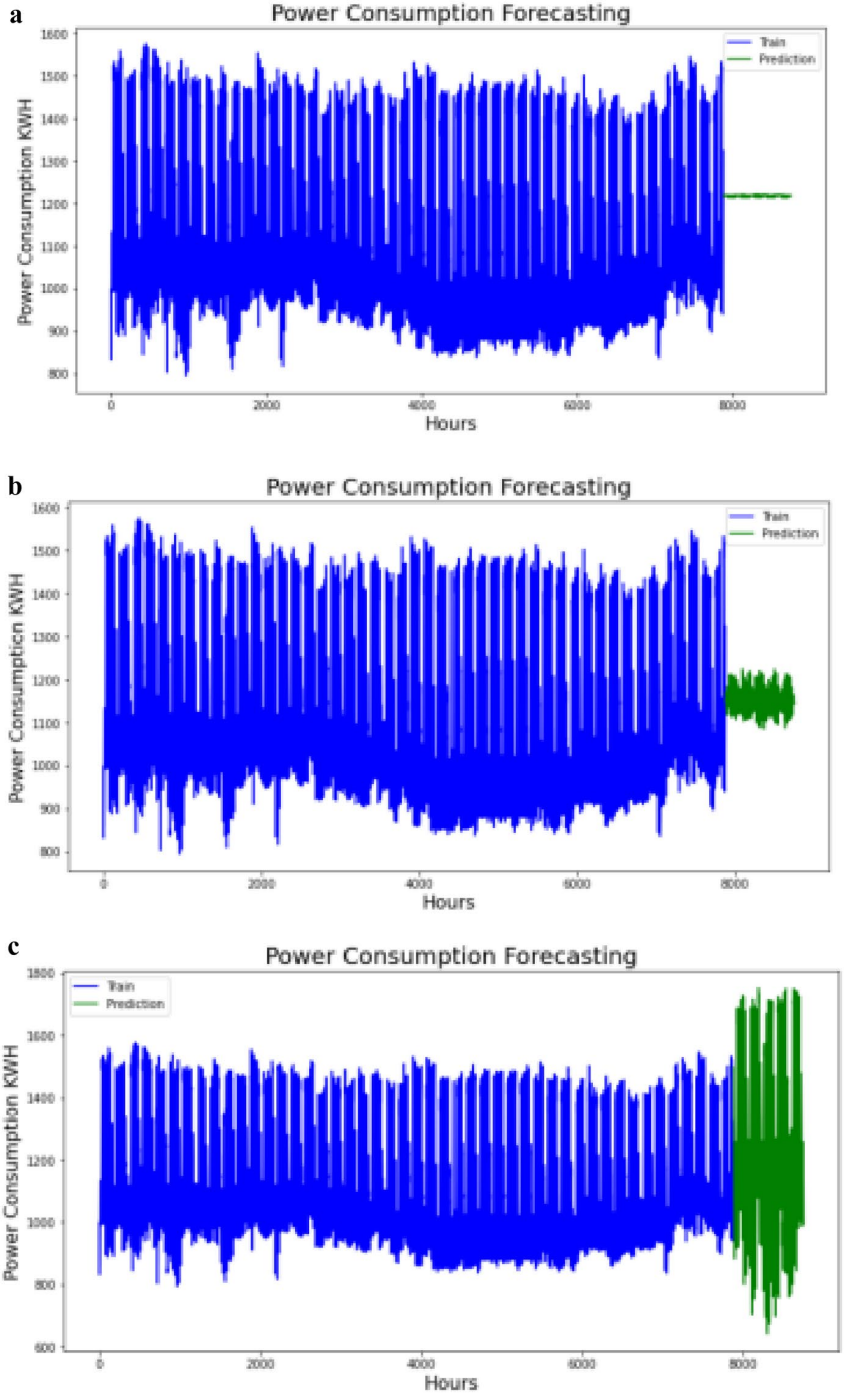

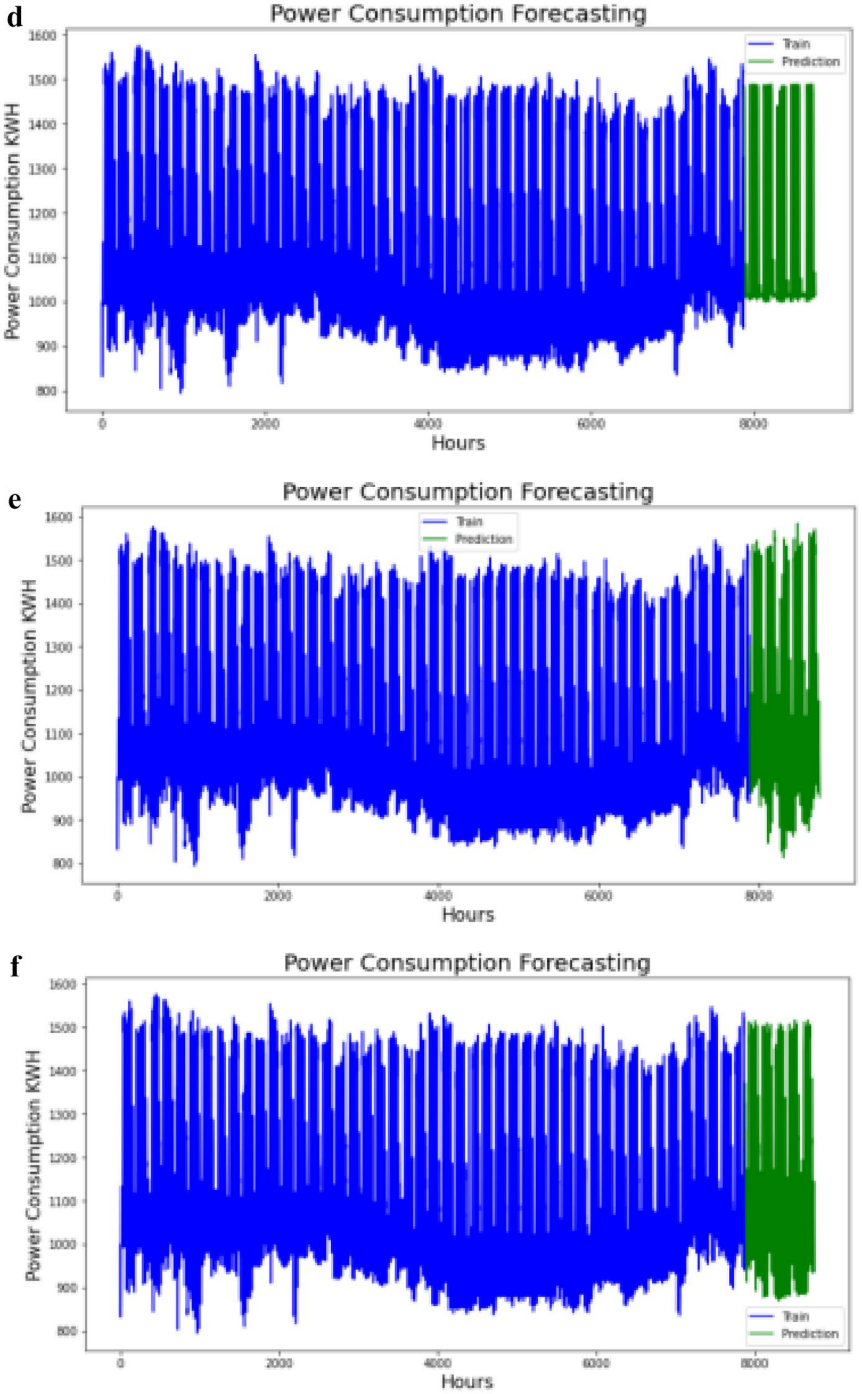
(**a**) Training data and forecasted values in step-1 setting. (**b**) Training data and forecasted values in step-2 setting. **(c**) Training data and forecasted values in step-3 setting. (**d**) Training data and forecasted values in step-4 setting. (**e**) Training data and forecasted values in step-5 setting. (**f**) Training data and forecasted values in step-6 setting.

After we have finalized and successfully trained our model, the power consumption values of the future time steps were then forecasted by using the model Predict() class in Keras model. In LSTM, the prediction of next time step value occurs one at a time and update the LSTM network at each prediction. The first prediction is made using the last observation of training. Subsequently, the previous predicted values were being used as input for next prediction steps. Figure 3a–f) shows the training data plots with forecasted values for multiple settings of hypermeters.

#### Comparison of test data with forecasted values

**Figure 4:**
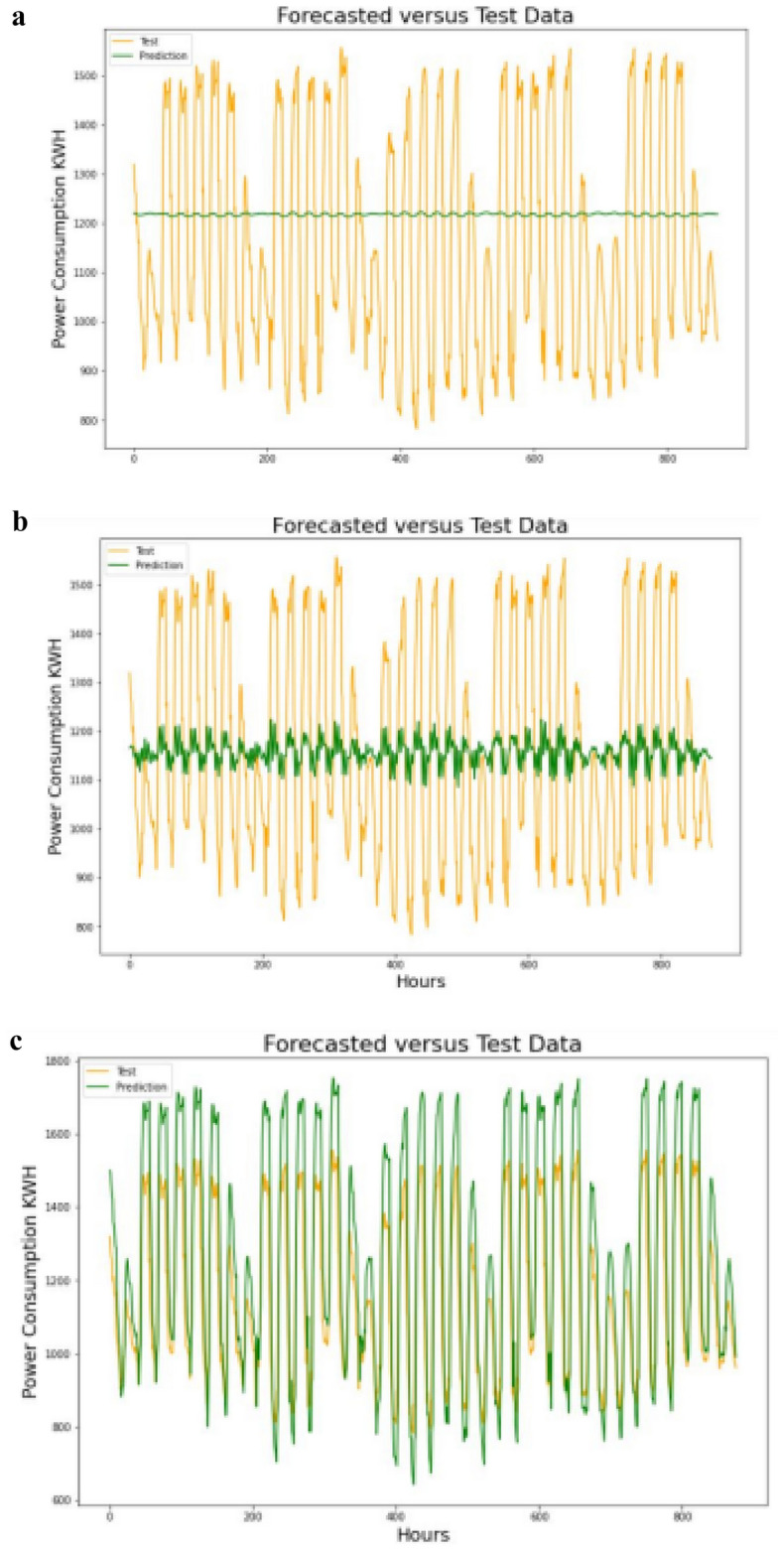

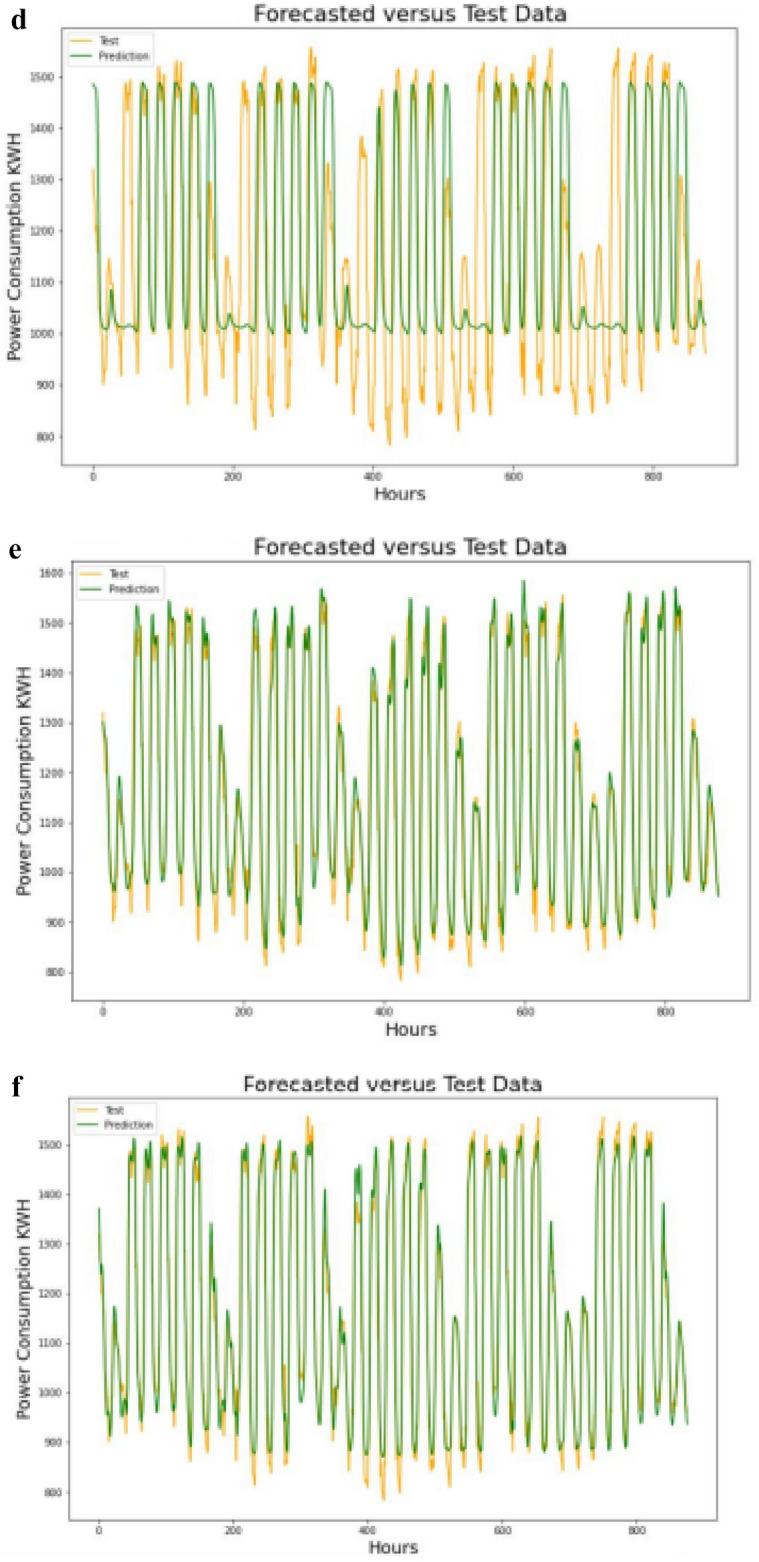
(**a**) Test data and forecasted values at step-1 setting. (**b**) Test data and forecasted values at step-2 setting. (**c**) Test data and forecasted values at step-3 setting. (**d**) Test data and forecasted values at step-4 setting. (**e**) Test data and forecasted values at step-5 setting. (**f**) Test data and forecasted values at step-6 setting.

The forecasted values obtained has then been plotted against the test data for different settings of hypermeters as shown in Fig. 4(a–f).

#### Optimizations result

As discussed earlier our objective function is to maximize the R-squared and conversely minimize the mean absolute loss. The accuracy that each setting obtained in terms of higher R-Squared and lower errors is reported in Table 4 below.

**Table 4:** Objective function results.

Cost function	Step-1	Step-2	Step-3	Step-4	Step-5	Step-6
R-2	− 5%	14%	46%	49%	93%	95%

## Conclusion

In this paper, we have primarily addressed the two significant issues of model optimization and electricity consumption forecasts. Future forecasting has been done using the LSTM based time series approach. The optimization was used to improve the algorithms' accuracy while, conversely, reducing their loss. We generated data on the amount of electricity consumed by a hospital facility and tested our suggested methodologies on actual data. The findings gained demonstrated that the strategies were successful with both types of data. On actual data, the trend in electricity consumption can be accurately predicted. Several model optimizers enhanced the suggested methods’ performance as well. In summary, researchers are now concentrating on discovering methods that could model their data and produce an accurate forecast in accordance with their application and limits, whether it be short-, medium-, or long-term load projections. This study will help researcher to find accurate analysis about future consumption of electricity. By using optimizers we gained good results and get improved algorithm accuracy. Our objective function is to maximize the R-squared and conversely minimize the mean absolute loss and gained 95% accuracy rate. This research can be enhanced by using large datasets of different organizations in order to get more accurate results.

## Data Availability

The datasets generated during and/or analyzed during the current study are available in the Shahid-Fakhri/Electricity-Consumption repository, [http://github.com/Shahid-Fakhri/Electricity-Consumption].
